# Modification of Poly(3-Hydroxybutyrate) with a Linear Polyurethane Modifier and Organic Nanofiller—Preparation and Structure–Property Relationship

**DOI:** 10.3390/ma17225542

**Published:** 2024-11-13

**Authors:** Iwona Zarzyka, Beata Krzykowska, Karol Hęclik, Wiesław Frącz, Grzegorz Janowski, Łukasz Bąk, Tomasz Klepka, Jarosław Bieniaś, Monika Ostapiuk, Aneta Tor-Świątek, Magda Droździel-Jurkiewicz, Adam Tomczyk, Anna Falkowska, Michał Kuciej

**Affiliations:** 1 Department of Organic Chemistry, Faculty of Chemistry, Rzeszow University of Technology, Powstancow Warszawy 6, 35959 Rzeszow, Poland; b.krzykowska@prz.edu.pl; 2 Department of Biotechnology and Bioinformatic, Rzeszów University of Technology, Powstancow Warszawy 6, 35959 Rzeszow, Poland; kheclik@prz.edu.pl; 3 Department of Materials Forming and Processing, The Faculty of Mechanical Engineering and Aeronautics, Rzeszow University of Technology, al. Powstancow Warszawy 12, 35959 Rzeszow, Poland; wf@prz.edu.pl (W.F.); gjan@prz.edu.pl (G.J.); lbak@prz.edu.pl (Ł.B.); 4 Department of Technology and Polymer Processing, Faculty of Mechanical Engineering, Lublin University of Technology, Nadbystrzycka 36, 20618 Lublin, Poland; t.klepka@pollub.pl (T.K.); a.tor@pollub.pl (A.T.-Ś.); 5 Department of Materials Engineering, Faculty of Mechanical Engineering, Lublin University of Technology, Nadbystrzycka 36, 20618 Lublin, Poland; j.bienias@pollub.pl (J.B.); m.ostapiuk@pollub.pl (M.O.); m.drozdziel@pollub.pl (M.D.-J.); 6 Faculty of Mechanical Engineering, Bialystok University of Technology BUT, 45C Wiejska, 15351 Bialystok, Poland; a.tomczyk@pb.edu.pl (A.T.); a.falkowska@pb.edu.pl (A.F.); m.kuciej@pb.edu.pl (M.K.)

**Keywords:** nanobiocomposites, linear chain polyurethanes, nanoadditives, modified montmorillonite, internal structure, mechanical properties

## Abstract

The growing demand for products made of polymeric materials, including the commonly used polypropylene (PP), is accompanied by the problem of storing and disposing of non-biodegradable waste, increasing greenhouse gas emissions, climate change and the creation of toxic products that constitute a health hazard of all living organisms. Moreover, most of the synthetic polymers used are made from petrochemical feedstocks from non-renewable resources. The use of petrochemical raw materials also causes degradation of the natural environment. A potential solution to these problems is the use of biopolymers. Biopolymers include biodegradable or biosynthesizable polymers, i.e., obtained from renewable sources or produced synthetically but from raw materials of natural origin. One of them is the poly(3-hydroxybutyrate) (P3HB) biopolymer, whose properties are comparable to PP. Unfortunately, it is necessary to modify its properties to improve its processing and operational properties. In the work, hybrid polymer nanobiocomposites based on P3HB, with the addition of chain, uncross-linked polyurethane (PU) and layered aluminosilicate modified with organic salts (Cloisite^®^30B) were produced by extrusion process. The introduction of PU and Cloisite^®^30B to the polymer matrix (P3HB) influenced the processing parameters beneficially and resulted in a decrease in the extrusion temperature of more than 10 °C. The influence of the simultaneous addition of a constant amount of PU (10 m/m%) and the different amounts of nanoadditives (1, 2 and 3 m/m%) on the compatibility, morphology and static mechanical properties of the resulted nanobiocomposites were examined. The component interactions by Fourier transformation infrared spectroscopy (FTIR) analysis, nano- and microscale structure studies using small-angle X-ray scattering (SAXS) and morphology by scanning electron microscopy (SEM) were carried out, and the hardness and tensile strength of the obtained polymer nanobiocomposites were determined. FTIR analysis identified the compatibility of the polyester matrix, PU, and organomodified montmorillonite, the greatest being 3 m/m% Cloisite30B content. The addition of PU to the polyester elasticizes the material and decreases the material’s strength and ductility. The presence of nanoclay enhanced the mechanical properties of nanobiocomposites. The resulting nanobiocomposites can be used in the production of short-life materials applied in gardening or agriculture.

## 1. Introduction

Plastics are ubiquitous in modern life due to their versatility, durability, and low cost. Because of their unique properties, they often replace other materials such as wood, glass, and metal. As a result of the rising standard of living in modern societies, rapid technological development and constantly increasing consumption, the demand for products made of polymer materials has increased dramatically.

One of the commonly used polymers from the polyolefin group is polypropylene (PP) [1]. Its share in the plastics market is the largest, approximately 19.2%, and is constantly increasing [2]. Thanks to its versatile application, it is widely used. It can be found both in households in the form of elements such as housing panels for refrigerators, washing machines and fans, but also in more demanding applications, e.g., in the automotive industry (car bumpers, dashboards, etc.), food industry (food packaging) and medical industry (laboratory equipment, equipment covers). As a result of the widespread use of PP in various sectors of the economy, the problem of a constantly increasing amount of waste, both post-consumer and production, has arisen [3,4].

At the same time, there has been a problem of increasing environmental pollution resulting from improper waste management, limited natural resources and the increase in crude oil prices [2,3,5]. An alternative to becoming independent from petroleum, while getting rid of tons of waste, has become material recycling and reusing plastic elements (regardless of the moment of the product life cycle) in less demanding applications [4,5].

Another alternative is biodegradable plastics with a much shorter degradation time. Any biodegradable polymers can be engineered to have mechanical properties that are comparable to petroleum-based plastics. Biopolymers have been found in many applications, including medicine, pharmacy and the packaging industry [6,7,8,9,10]. As interest in sustainability and environmental conservation grows, the development and adoption of biodegradable polymers represent a promising direction for reducing plastic waste and pollution [11,12].

Poly(hydroxyalkanoates) (PHAs) are biosynthesizable and biodegradable and therefore have a particular importance in the group of biopolymers [13]. Poly(3-hydroxybutyrate) (P3HB) is one of the most representative PHAs [14,15]. P3HB is synthesized and stored as reserve material in microbial cells [16]. The P3HB properties such as the degree of crystallinity, some mechanical properties and the range of melting and glass transition temperature values are comparable to the properties of isotactic PP [17].

P3HB is biocompatible and has a lack of cytotoxicity and mutagenicity [18,19,20,21]. However, P3HB has some defects; it is a stiff and fragile material and has a low degradation temperature. Therefore, P3HB is often subjected to numerous modifications to improve its properties and expand the scope of applicability of this material [22,23,24]. The production of compositions based on P3HB or composites with the addition of polymer modifiers leads to the desired change in its properties and processing conditions, because of the favorable separation of the melting temperature and the degradation temperature [25,26,27,28]. One way of the P3HB properties modification is the preparation of the hybrid composites or nanocomposites with the use of nanoadditives, e.g., nanotubes as reactive additives [29] or physical additives or other nanoadditives [30].

In recent years, polymer nanocomposites with nano-addition of aluminosilicates have attracted wide interest due to significant improvement of polymer properties using small amounts of nanofiller [6,31]. Numerous studies have been conducted with the addition of layered silicates such as saponite, bentonite, kaolinite and montmorillonite (MMT) [32,33]. Montmorillonite belongs to smectic clays. It is composed of silicate platelets with an internal octahedral layer placed between two tetrahedral layers [34]. Its unique advantages make it suitable for improving the material properties. Usually, montmorillonite modified with quaternary ammonium cations is used. In this way, the gap from layered silicates is increased by the ion exchange reaction of Na^+^ ions present in natural montmorillonite with various organic cations of amine type [35]. Polymer nanocomposites containing organically modified montmorillonite clays such as Cloisite 10A, 15A, 20A, 30B and Cloisite 93A exhibit better thermal, physicochemical and mechanical properties compared to the native polymer [36,37]. One of the most commonly used organically modified nanoclays is Cloisite^®^30B, e.g., polylactic acid bionanocomposites exhibit better thermal stability in the case of Cloisite30B compared to other types of montmorillonite such as Cloisite Na^+^, Cloisite 20A and Cloisite 25A [38].

In the work, the hybrid polymer nanobiocomposites based on poly(3-hydroxybutyrate) with the addition of other polymer modifier-linear, chain polyurethane synthesized in the reaction of a 1,6-hexamethylene diisocyanate and polypropylene glycol with a molar mass of 400 g/mol and organically modified montmorillonite-Cloisite^®^30B were produced. The miscibility, nano- and micro-structure, and some mechanical properties of the resulting nanobiocomposites were examined and their properties were assessed in terms of the granulate production suitable to prepare biodegradable products, e.g., agrofabric.

## 2. Materials and Methods

### 2.1. Materials

P3HB was purchased from Biomer (Krailling, Germany); the Mw of P3HB was 400,000 to 1,200,000 g·mol^−1^ and its dispersity index was 5.72; the melt flow index of P3HB has a level of 0.11 g·(10 min)^−1^, 180 °C at 2.16 kg. Organically modified montmorillonite by methylbis(2-hydroxyethyl)tallowalkyl-ammonium cations, commercially called Cloisite^®^30B, was purchased from Southern Clay Products Inc. (Gonzales, LA, USA). Cloisite 30B contains methyl tallow bis(2-hydroxyethyl)ammonium cations, at a loading of 90 meq/100 g clay (Figure 2), where tallow is app. 65% C_18_H_37_; app. 30% C_16_H_33_; app. 5% C_14_H_29_. In addition, 1,6-Hexamethylene diisocyanate (HDI) and polypropylene glycol with molecular weight 400 g·mole^−1^ (PPG400), dibutyltin dilaurate (DBTL) were supplied by Aldrich (Darmstadt, Germany), and acetone by Chemsolute (Aesch, Switzerland).

### 2.2. Preparation of Linear Polyurethane

Anhydrous PPG400, anhydrous acetone and DBTL in an amount of 0.003 mol·mol^−1^ PPG400 were placed in a three-necked round-bottom flask with a flow of nitrogen. The solution was mixed by a mechanical stirrer. HDI was added from a dropper to the solution PPG400 in acetone. PPG400 and HDI were used in such a molar ratio that the molar ratio of hydroxyl groups to isocyanate groups was 1:0.93. The rate of HDI instillation depended on the temperature of the reacting mixture which was smaller than 20 °C. Synthesis was monitored by control of the isocyanate group content (ICN). When ICN was 0, the reaction was finished and the acetone was removed in the rotary evaporator. The residual acetone was removed in a vacuum dryer at a temperature in the range of 40–100 °C. The hydroxyl number of PU is about 5 mg KOH·g^−1^. Molar masses of PU are presented in Section 2.1.

### 2.3. Composite Production

P3HB and 10 m/m% of PU and the nanoadditive-organomodified montmorillonite in the amount of 1–3 m/m% were extruded on a twin-screw extruder, type. RES-2P12A Explorer by ZAMAK-Mercator (Skawina, Poland). The use of the increasing content of Cloisite^®^30B did not cause any changes in the extrusion temperature. The bionanocomposites were prepared with the use constant temperature profile of zones in the twin-screw extruder, as shown in Table 1.

Keeping the extruder charging temperature within 50–55 °C is crucial to control the viscosity of the material. Variations of the temperature outside this range have significant effects, necessitating strict temperature monitoring and control to avoid operational issues.

Lower temperatures lead to higher loads on the system, while higher temperatures decrease material viscosity. This could complicate the cooling and granulation processes. The temperature profile is shown in Table 1. The temperature of zones number 3–7 must be constant because the processing window of the produced nanobiocomposites is very tight.

Continuous monitoring of the instantaneous load on the drive system is vital. Any increases approaching the disconnection threshold (110% of nominal load) should trigger adjustments to avoid overloading.

Operating the screw at 80–120 rpm strikes a balance, but adjustments may be necessary based on the specific load and temperature readings to optimize flow without exceeding the drive thresholds (Table 2).

The processing of produced hybrid nanobiocomposites containing 1–3 m/m% Cloisite^®^30B is similar to the processing of composition P3HB with 10 m/m% of PU (series 01). After leaving the extrusion head, the extrudate must be thoroughly cooled because it has a very low viscosity. Intensive cooling of the extrudate must be carried out right next to the extrusion head. The extrudate was placed in a cooling bath with cold water, which ensured the possibility of granulating the extrudate. The small content of PU (10 m/m%) makes the extrusion temperature of the composition, compared to native P3HB, significantly lower.

A higher content of Cloisite^®^30B does not affect the temperature and extrusion temperature profile. There was no need to modify the extrusion parameters. After production, the extrudate (strand with a diameter of approx. 2–3 mm) is not very flexible and allows for an easy mechanical granulation process. After reaching room temperature (completely cooled), the extrudate is moderately elastic.

Granulation of extrudate was carried out with the use a G1of 6/32-II granulator from Zamak-Mercator (produced by ZAMAK Mercator company, Skawina, Poland).

### 2.4. Preparation of Samples for Testing Mechanical Properties

Samples for testing the mechanical properties of the obtained material were produced using a Dr BOY 55E injection molding machine (produced by BOY Maschines Inc., Exton, PA, USA). The processing parameters presented in Table 3 were used during the injection process.

The setting temperature of the plasticization system, depending on the amount of PU, is shown in Table 4.

The processing parameter settings were established by analyzing the pressure profiles within the injection mold cavities during each production cycle, utilizing data recorded by the Priamus system.

During the production of samples, it was found that the material could not be injected into a cold mold or into a hot mold at a temperature above 80 °C, because the molded pieces contain large shrinkage cavities. The low thermal stability of the material does not allow it to be kept longer in the plasticizing cylinder of the injection molding machine. All interruptions in the process and even longer plasticization processes result in thermal degradation of the material.

### 2.5. Methods

#### 2.5.1. Determination of Hydroxyl Number

The hydroxyl number was measured according to the standard [39].

#### 2.5.2. Determination of Isocyanate Group Content

Content of isocyanate groups (ICN) in % mass were marked according to the standard [40].

#### 2.5.3. Determining the Molar Mass Characteristics of Linear Polyurethane

The process for determining the molar masses characteristic of linear polyurethane was made using High-Temperature Gel Permeation Chromatography (HT-GPC). A dual detection setup was employed, involving both refractive index and viscosity detectors. Dimethylformamide (DMF) was used as the solvent and a sample concentration of approximately 3 mg/mL. A sample injection volume of 100 μL was used. A combination of PL gel columns was used for separation: 1 × Mixed-A (300 × 7.8 mm, 15 μm particles), 1 × Mixed-B (300 × 7.8 mm, 10 μm particles), 1 × Mixed-D (300 × 7.8 mm, 5 μm particles). The analysis was performed at 50 °C. The flow rate during the analysis was maintained at 1.0 mL/min. The GPC system was calibrated using polystyrene standards in the range 580 to 271,000 g/mol (Polymer Laboratories Ltd., Church Stretton, UK) using 12 calibration points for universal calibration. Cirrus software (https://cirrussoftware.com/, accessed on 7 November 2024) was used for processing the data collected during the GPC analysis.

#### 2.5.4. FTIR Measurements

The FTIR spectrum of P3HB was recorded as a KBr pellet, while the polymer composition and hybrid nanobiocomposites were analyzed using the ATR technique with the ALPHA FT-IR instrument from BRUKER (Karlsruhe, Germany). Spectra were collected in the wavenumber range of 400 to 4000 cm^−1^, and a resolution was 2 cm^−1^.

#### 2.5.5. SAXS Measurements

To investigate the nanoclay structure in the prepared biocomposites, the small-angle X-ray scattering (SAXS) technique was utilized within the 1 to 10° range. The measurements were made at room temperature with the use of a Bruker SAXS Nanostar-U X-ray diffractometer. Sample spectra were analyzed in transmission mode. The SAXS apparatus is connected to a filtered CuKa radiation source (1.54 Å) in a sealed tube, operating at 50 kV and 30 mA. A 2D detector (Vantec2000) was employed to scan the entire surface of the samples, with a beam spot size of approximately 500 μm. The scanning range was adjusted by varying the distance between the sample and the detector. With a resolution that allows for 2048 × 2048 pixel measurements, the detector covered an angular range from 1 to 10° over a duration of 1 h.

#### 2.5.6. Mechanical Tests

The hardness of the tested materials was determined with the use of a Shore hardness tester (Zwick/Roell, Ulm, Germany) in accordance with ISO 868 [41]. In the case of the tested materials, the obtained values of strength characteristics (especially the value of Young’s modulus) allow them to be classified as hard materials. Therefore, a D-type hardness tester was used, where the indenter is a steel rod with a diameter of 1.1–1.4 mm, with a conical tip, rounded with a radius of 0.1 mm (cone angle of 30°). Hardness was measured both in the specimen gauge length (zone I) and in the gripped part (zone B). Both zones are shown schematically in Figure 5. In an attempt to determine the hardness, we decided not to use the Brinell method due to the too-narrow area in the specimen gauge length. During tests of pressing the Brinell indenter, significant damage to the material occurred due to the short distance of the specimen edge from the place where the indenter was pressed.

Monotonic tensile tests were carried out with the use of a biaxial, servo-hydraulic MTS 858 Mini Bionix system (Maumee, OH, USA) with an axial load range of ±25 kN and a torsional load range of ±250 Nm. An Instron 2620-601 axial extensometer with a base of 75 mm and a range of ±5 mm was used to measure longitudinal strains. Due to the need to determine the Poisson’s ratio *ν*, an Instron 632.18F-20 transverse extensometer (Norwood, MA, USA) with a base of 10 mm and a range of ±2.5 mm was also used. Transverse strains were measured on the longer side of the sample cross-section. Experimental investigation aimed at determining the basic strength characteristics of the newly produced nanocomposites, i.e., Young’s modulus *E*_t_, Poisson’s ratio *ν*, yield strength *σ*_y_ (if it exists), ultimate tensile strength *σ*_M_ and the corresponding strain *ε*_M_, strength at break *σ*_B_ and the corresponding deformations *ε*_B_. The tests were performed according to the ISO 527-1 standard [42]. The tensile test of one sample from each series was also recorded using the Aramis 3D 4M digital image correlation system. It was used to determine changes in the stress and strain distribution of the specimen gauge over time for the chosen series. In the case of the materials that do not have a *σ*_y_ limit according to the relevant standard, the stress *σ*_0.01_ corresponding to the strain *ε* = 0.01 was determined in order to compare the results achieved for different series.

#### 2.5.7. Scanning Electron Microscopy Measurements

The fracture surfaces observed after the notch impact tests were analyzed by a scanning electron microscope SEM (NovaNanoSEM 450, FEI Company Japan Ltd., Tokyo, Japan) by applying a low vacuum mode of 100 Pa and voltages of 10 and 15 keV. The secondary electron technique was used for the analysis of all samples. The spot size was 4.0 and 4.5. Samples were not coated with any chemical elements like chromium or gold.

## 3. Results and Discussion

### 3.1. Nanobiocomposites Based on P3HB

**Figure 1:**
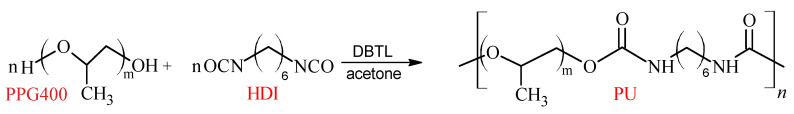
Synthesis scheme of polyurethane (PU).

To modify the properties of poly(3-hydroxybutyrate) (P3HB), a chain and uncross-linked polyurethane (PU) modifier was used. PU was synthesized in the reaction of polypropylene glycol with a molar mass of 400 g (PPG400) and 1,6-hexamethylene diisocyanate (HDI) which is shown in Figure 1. Nanocomposites were prepared with the use of PU in the amount of 10 m/m% and a nano-additive in the form of organo-modified aluminosilicate-Cloisite^®^30B in the amount of 1, 2 and 3 m/m%.

The use of a polyurethane modifier and a nano-additive should allow for improvement in the process (thermal) and mechanical properties of matrix material without worsening its biodegradation, and may even accelerate the biodegradation of the new product. It is known that the presence of hydrophilic polymers increases the water retention in the polymer blends and accelerates their hydrolysis (biodegradation) [25].

The molar masses of the used PU are shown in Table 5. The molar masses are relatively low compared to those of P3HB. Mw of P3HB was 400,000 to 1,200,000 g·mol^−1^ and its dispersity index was 5.72. The molar mass of PU–M_w_ = 26,850 g mol^−1^ indicates a degree of polymerization of about 47. Relatively short polyurethane chains should make it easier to separate long polyester chains, reduce their interactions and cause a flexibilizing effect. Bakar et al. described this [43] phenomenon as the lower molar mass of the polymer modifier, the better the plasticizing effect observed.

In order to compare the effect of the PU modifier and nanoadditive on the properties of P3HB, native P3HB was also subjected to the extrusion process and a P3HB polymer composition with 10 m/m% of PU (series 01).

The processing of a native P3HB can take place at temperatures much lower than 180 °C. The addition of PU plasticizer to P3HB further reduces this temperature to approximately 175 °C. This allows for lower energy costs related to the plasticization of the plastic or polymer composites. Therefore, in terms of energy demand, P3HB with its modifications using PU is a more ecological material than polypropylene (PP) whose processing is usually carried out at temperatures above 190 °C.

Another issue is the extrusion of profiles from pure PP or polymer composites based on PP, which requires the use of water cooling of the extrudate. In the case of P3HB polymer compositions and composites, cooling is carried out in the open air. This also reduces the energy expenditure associated with energy demand during processing.

Lower temperatures of P3HB-PU-based composite processing allow the use of fillers whose degradation temperature is low and often cannot be added to PP due to the risk of their degradation. Filler degradation results in gases released during processing and it disturbs the processing conditions and causes various defects in the products.

### 3.2. FTIR Analysis of the Resulted Biocomposites

**Figure 2:**
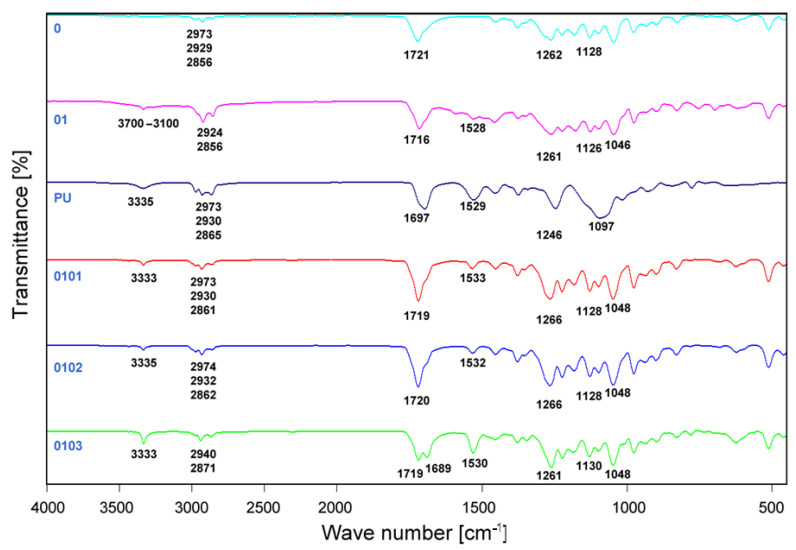
FTIR spectra of native P3HB (series 0), P3HB-PU (series 01) composition, PU and produced hybrid biocomposites containing 10 m/m%. PU and 1, 2, or 3 m/m% Cloisite^®^30B designated as series 0101, 0102, 0103.

FTIR characterization of P3HB, PU, P3HB-PU compositions (series 01) and nanobiocomposites based on P3HB-PU was performed to check possible interactions between the ingredients of the biocomposites [44]. The FTIR spectra of P3HB (series 0) and PU, P3HB-PU (series 01) composition and hybrid nanobiocomposites with different Cloisite^®^30B clay content (series 0101, 0102 and 0 103) are shown in Figure 2. For the purpose of this paper, material nomenclature was introduced in Table 2 in the Section 2.

FTIR spectrum of a native P3HB (series 0) showed characteristic peaks, i.e., strong absorption at 1721 cm^−1^, indicative of valence vibration of C=O ester bond and a band of asymmetric and symmetric vibrations C-O bonds at 1262 cm^−1^ and 1128 cm^−1^. In the FTIR spectrum of PU, there is a notable peak at 3335 cm^−1^ from the valence vibration of N-H bonds of urethanes. Furthermore, we can observe peaks at 2865 cm^−1^, 2930 cm^−1^, and 2973 cm^−1^ characteristic of C-H bonds of methylene groups, a peak at 1697 cm^−1^ characteristic of C=O bond in urethane groups, and a peak at 1529 cm^−1^. from N-H deformation vibrations, and peaks at 1246 cm^−1^ and 1097 cm^−1^ from C-O vibrations in urethane groups.

A wide band observed in the range of 3700–3100 cm^−1^ in the FTIR spectrum of series 01 biocomposition signifies possible interactions between urethane groups and ester groups through hydrogen bonding. A peak at 1716 cm^−1^ derived from C=O stretching vibrations indicates interactions between ester and urethane functionalities. Detected in the range of 1585–1650 cm^−1^, bands from N-H bending vibrations suggest intermolecular hydrogen bonding formation between the P3HB and PU chains [45,46]. The spectra revealed also asymmetric and symmetric C-O vibrations at 1261 cm^−1^, 1126 cm^−1^, and 1046 cm^−1^.

**Figure 3:**
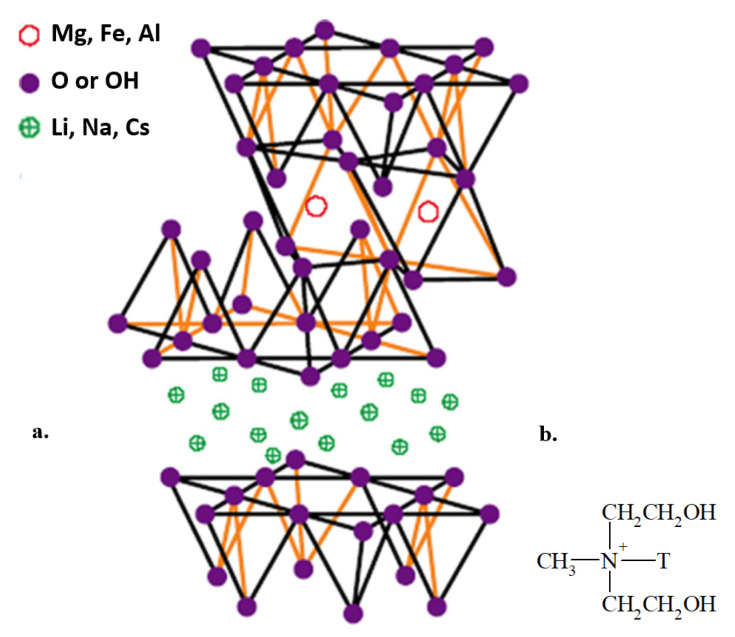
Schematic structure of montmorillonite (**a**), structure of tallow ion (**b**). Where T—tallow group is app. 65% C_18_H_37_; app. 30% C_16_H_33_; app. 5% C_14_H_29_.

**Figure 4:**
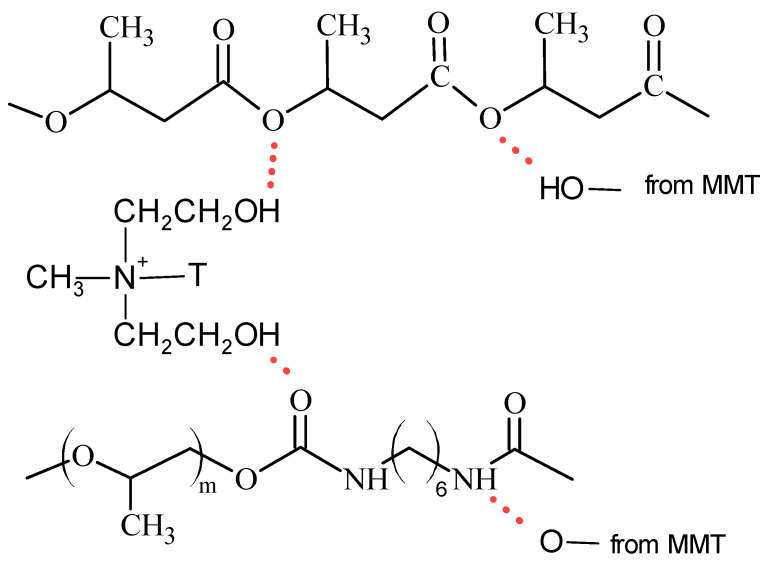
Scheme of nanobiocomposites components interactions by hydrogen bonds.

In the case of FTIR spectra of hybrid nanobiocomposites with Cloisite^®^30B (structure Cloisite^®^30B is shown in Figure 3), increased intensity of the band at 3335 cm^−1^ with higher Cloisite^®^30B content, peaking at 3 m/m%, indicated enhanced interaction among composite components, as is illustrated in Figure 4.

The hydrophilic nature of montmorillonite prevents the penetration of polymer chains between its layers. Hydrophobization of MMT consisting of the introduction of quaternary ammonium cations instead of interlayer cations leads simultaneously to an increase in the distance between the aluminosilicate layers and an increase in its affinity for the polymer matrix [47,48,49].

The introduction of Cloisite 30B into the P3HB-PU matrix causes an increase in intermolecular interactions, including the formation of hydrogen bonds between the organically modified aluminosilicate and the polyester and polyurethane chain, as well as van der Waals interactions.

The narrower band width in nanobiocomposites compared to the P3HB-PU composition implies reduced interaction between PU and P3HB. The band at 1719 cm^−1^ attributed to C=O stretching is split into two distinct bands when 3 m/m% of PU is present, indicating strong interactions. Higher intensity of N-H bending vibrations between 1533–1530 cm^−1^ in the nanocomposite suggests significant intermolecular hydrogen bond formation [45,46].

The FTIR analysis confirms effective interactions between PU, Cloisite^®^30B, and the P3HB matrix, especially pronounced in the biocomposite with 3 m/m% Cloisite^®^30B (series 0103). This detailed analysis provides a comprehensive understanding of the complex interactions within the biocomposite system. This study is a crucial step in understanding the compatibility and enhancement of properties in biopolymer blends through nanoadditives, indicating potential applications in various fields, including biomedical materials and sustainable materials for agriculture.

### 3.3. Nanostructure Analysis of P3HB Biocomposites

**Figure 5:**
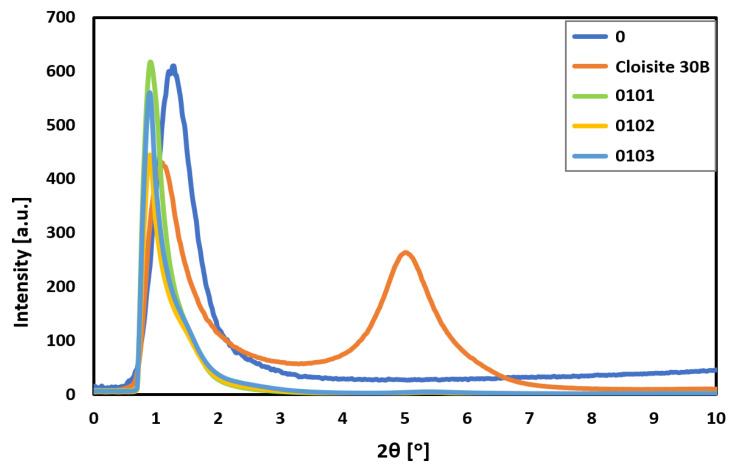
SAXS plots of native P3HB (0), Cloisite^®^30B, and produced hybrid biocomposites containing 10 m/m%. PU and 1, 2, or 3 m/m% Cloisite^®^30B designated as 0101, 0102, 0103.

In turn, the nanostructure of biocomposites was studied. A nanoadditive dispersion in the polymer matrix was checked by SAXS measurements and the results are shown in Figure 5. The plot of the produced biocomposites is presented, as well as the plots of native P3HB and the plot of Cloisite^®^30B nanoclay for comparison. Native P3HB only exhibits background scattering below 10°, indicating that it does not show significant layered or crystalline structure at the nanoscale in this range. The diffraction pattern of Cloisite^®^30B shows a peak at approximately 5.00° (2θ), corresponding to an interlayer spacing of around 1.77 nm. This peak suggests the presence of ordered arrangements of aluminosilicate layers typical of layered silicates. In the hybrid biocomposites produced, the absence of a peak in the diffraction pattern below 5° indicates that the Cloisite^®^30B has been completely delaminated. The polymer chains of polyester and polyurethane appear to have infiltrated the spaces between the aluminosilicate layers, disrupting their organization and leading to complete exfoliation. The complete delamination of Cloisite^®^30B signifies a shift from a layered structure to an exfoliated structure within the nanocomposites. This change is crucial because the exfoliation likely enhances the material properties significantly compared to the native polyester. The dispersion of nanoclay at the nanoscale can lead to improved mechanical strength, thermal stability, and other beneficial properties in the hybrid materials.

### 3.4. Mechanical Properties of the Resulted Bionanocomposites

#### 3.4.1. Hardness of P3HB-PU Based Nanobiocomposites

**Figure 6:**
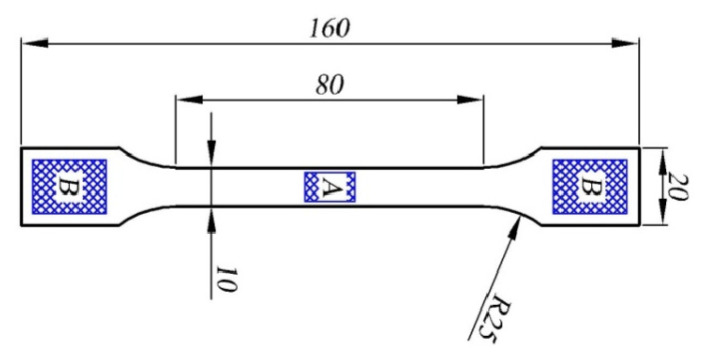
Shore hardness specimen with zones A and B (dimensions in mm).

Selected mechanical properties of the hybride nanobiocomposites were tested. The hardness test results were averaged and shown in Table 6. A single result was the average of three measurements in the same zone (*A* or *B*) of the sample. The obtained results allow us to conclude that the base material is characterized by the highest hardness. It should be noted that the addition of PU to the base material (series 01) causes a significant decrease in hardness, while the addition of clay (series 0101, 0102, 0103) increases it again. A similar tendency is also observed in the case of strain at break *ε*_B_, *ε*_fB_ and stress at break *σ*_B_, *σ*_fB_ (see Table 7). In the case of the modified material, the highest parameters were obtained for series 0101. This material is also characterized by the smallest difference in hardness measurement in the measuring part of the sample and in the gripping part. It is worth noting that the hardness of the material in the gripping part of the specimen (zone *B*) is always greater than the hardness in the gauge length (zone *A*), (Figure 6). This does not apply to the base material where the hardness in both zones is identical.

#### 3.4.2. Monotonic Tension of P3HB-PU Based Nanobiocomposites

The monotonic tension tests were made on five specimens of one series and the test results were averaged. The standard deviation *s* was determined for each of the determined values. What is noteworthy is the repeatability of the results within each series, except for series 0102, where the scatter is always the largest (Table 7). At the same time, within each series, the largest differences concern the strain at break *ε*_B_. However, such regularity can be observed not only for polymeric materials but also for metals and their alloys. The value of the strain at break is often largely determined by local imperfections in the shape of the sample or the microstructure of the material itself. The material of series 01, 011, 012 and 013 is a more brittle material and at the same time less stiff than the base material 0.

The results of the tests show that the series 0–P3HB belongs to the group of hard materials with a yield strength of *σ*_y_. In the analyzed case, we have *σ*_y_ = *σ*_M_. All other materials belonging to the series resulting from modification of the base material are hard materials that do not have a *σ*_y_ limit within the meaning of the relevant standard and then *σ*_B_ = *σ*_M_.

The base material (series 0) has the best strength properties (Table 7). The stiffness of the materials of series 01, 0101, 0102 and 0103 is much lower than that of the base material. The difference is approximately 24%. This, of course, results in an increase in the Poisson’s ratio of materials from the mentioned series in relation to the base material. Both the values of the *E*_t_ module and the *ν* ratio for series 0101, 0102 and 0103 remain basically at a similar level. The noticeable differences in this series concern mainly the stress and strain at break (*σ*_B_, *ε*_B_). We note a significant decrease in both values for material from series 0103. In the case of series 01, 0101 and 0102, these values were higher. This applies especially to *ε*_B_. The composition of the P3HB with polyurethane (series 01) causes a substantial decrease in the ductility of the polyester. However, the presence of clay has the effect of increasing the ductility again. This effect is greatest for the lowest percentage of clay, i.e., for series 0101.

### 3.5. Fracture Analysis by Scanning Electron Microscopy (SEM)

**Figure 7:**
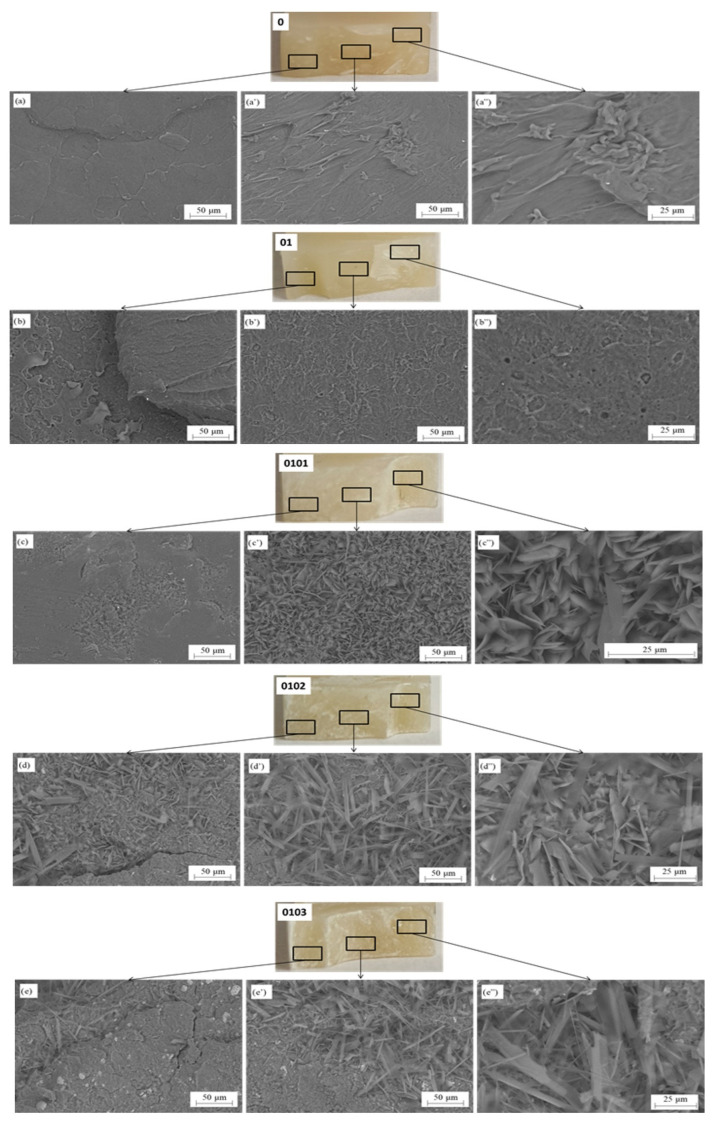
Fracture surface analysis of native P3HB (series 0), P3HB-PU (series 01) composition, and produced hybrid biocomposites containing 10 m/m% PU and 1, 2, or 3 m/m% Cloisite^®^30B designated as series 0101, 0102, 0103, after hardness test. (**a**–**e**)—left corner of sample, (**a′**–**e′**)—inside of sample, (**a″**–**e″**)—right corner of sample.

Figure 7 illustrates the SEM images of the fracture surfaces made for P3HB and biocomposition containing 10 wt.% of PU modifier (series 01) and biocomposites containing 10 m/m% of PU 1, 2 and 3 m/m% Cloisite^®^30B nanoadditive (series 0101, 0102, 0103). The SEM analysis after the hardness test was performed by SEM microscopy. The fracture surface of samples after the hardness test with names of the samples was viewed with macrophotography and microstructure. It was divided into three different zones: in two corners and inside the samples to observe the mechanism of destruction of materials. In relation to Figure 7a–a″, differences in fractures can be seen. This is caused by the elasticity of the material and the place where the force is applied. In addition, two areas are shown, characteristic of P3HB and 10 m/m% of PU without any additive, which can occur when this material breaks. In Figure 7a, the material shows a fairly smooth zone, while in Figure 7a″, a ductile zone is clearly visible.

Generally, the P3HB material after the test demonstrates the fracture surface to be polymeric zones. It was observed the characteristic plastic zones with riverlines. It was observed that the failure of the base sample (series 0) is of a brittle nature, as confirmed by the strength test results (Table 7). This material has a significantly higher stiffness in relation to the modified samples (0101, 0102, 0103), whose failure is of a more ductile nature. As shown for specimens (series 01), there was a significant decrease in the ductility of P3HB. The addition of clay increased the ductility of the 0101, 0102 and 0103 series material compared to the 01 series. In sample 01, the small porosities were visible where the 10% of PU was added. The adhesion zones on the surface and riverlines are in the outside region. The plastic deformation is in the center of the sample. For the modified samples, a different failure was observed with a rougher and more irregular fracture pattern. The other samples showed configuration with Cloisite^®^30B, and a completely different structure than the previous could be observed where P3HB was dominated material. In addition, the % of Cloisite^®^30B gave other results on the structure, but more the biodegradation started. The crystallites are similar to the corrosion on metals, so it could be thought that the biodegradation mechanism started. The crystallites possess a similar dimension, especially in 0101 samples. Moreover, the crystallites are in the whole surface after testing in three different regions. Materials 0102 and 0103 have similar fracture surfaces where there are zones with visible degradation of the P3HB. It could be observed that the different directions of the crystallites were situated in a polymeric matrix. The specific name for the crystallite could be “cold crystallite” which is characteristic not for the notch impact test but for i.e., humidity or another degradable condition which influences the samples. Interestingly, the samples with Cloisite^®^30B have white concentrated powder on the surface, which could prove that the biodegradation starts.

## 4. Conclusions

The study shows the production of P3HB-PU nanobiocomposites by an extrusion process with different concentrations (1–3 m/m%) of organomodified montmorillonite (Cloisite^®^30B) with the constant concertation of PU (10 m/m%).

The processing of a composition P3HB-PU takes place at temperatures much lower than a native P3HB, i.e., approximately 170 °C and lower than other popular but undegradable polymers, e.g., polypropylene, to which the properties of P3HB are very similar.

Spectral analysis confirmed successful P3HB/PU/montmorillonite interactions, with the optimal effect achieved at 3 m/m% Cloisite30B. This suggests strong interfacial adhesion.

X-ray analysis indicated the exfoliated structure of the resulting biocomposites and validated good nanofiller dispersion. This guarantees the best physicochemical and functional properties of the final material.

Regarding the mechanical properties of newly produced nanobiocomposites, it should be stated that they have worse strength properties than the native P3HB. The addition of PU to the base material causes a significant decrease in the material’s strength and ductility (series 01). In turn, the addition of nanoclay (series: 0101, 0102, 0103) causes another improvement in the mechanical characteristics. This applies in particular to tensile strength as well as parameters determining the stiffness of the material. A tendency to decrease the strength parameters with an increase in the organic nanoclay content in the resulting biocomposites was observed. At the same time, the hardness of nanobiocomposites becomes lower with an increased concentration of the modified montmorillonite.

The betterment of the mechanical and process parameters of the native poly(3-hydroxybutyrate) allows the application of the resulting nanobiocomposites to the production of short-life materials for gardening or agriculture.

## Figures and Tables

**Table 1:** Temperature of extruder zones during the production of the P3HB-based biocomposites with the addition of polyurethane modifier (10 m/m%) and the nanoadditive of Cloisite^®^30B in the amount of 1–3 m/m%.

Sample	T_Feed/Throat_ [°C]	T_1_ [°C]	T_2_ [°C]	T_3_ [°C]	T_4_ [°C]	T_5_ [°C]	T_6_ [°C]	T_7_ [°C]	T_Head_ [°C]
composites	50	145	155	163	163	163	163	163	163
P3HB	55	145	150	152	152	152	152	152	152

**Table 2:** Material nomenclature used in the paper.

Material Composition	Series Symbol
PH3B	0
PH3B + 10%PU	01
PH3B + 10%PU + 1% Cloisite^®^30B	0101
PH3B + 10%PU + 2% Cloisite^®^30B	0102
PH3B + 10%PU + 3% Cloisite^®^30B	0103

**Table 3:** Injection moulding parameters of samples prepared to test the mechanical properties, i.e., P3HB-based biocomposites with additive of 10 m/m% of PU and 1–3 m/m% of Cloisite^®^30B.

Parameter	Value
Filling Rate [cm^3^/s]	40
Filling pressure [bar]	550–600
Packing pressure (profile) [bar]	300–350
Packing time (profile) [s]	23
Mold temperature [°C]	40
Plasticization pressure [bar]	150

**Table 4:** The set temperature profile on the injection molding machine used for processing P3HB-based nanobiocomposites with 10 m/m% of linear PU and 1–3 m/m% of nanoclay–Cloisite^®^30B.

Series	T_1_ [°C]	T_2_ [°C]	T_3_ [°C]	T_4_ [°C]	T_Nozzle_ [°C]
0	170	174	177	179	180
01	170	173	175	178	178
0101	168	172	176	177	177
0102	168	172	174	177	177
0103	168	172	174	177	177

**Table 5:** Molar masses and dispersion degree of the obtained polyurethane.

M_n_ [g·mol^−1^]	M_w_ [g·mol^−1^]	M_p_ [g·mol^−1^]	DI
22,360	26,850	27,900	2.09

M_n_—number-average molar mass, M_w_—weight-average molar mass, M_p_—molar mass at peak maximum, and DI—the degree of dispersion.

**Table 6:** Averaged values of hardness (Shore D) for investigated materials.

Series	Zone *A* [ShD]	Zone *B* [ShD]
0	76	76
01	65	65
0101	70	71
0102	68	70
0103	68	71

**Table 7:** Values of basic strength characteristics for investigated materials.

Series	*E*_t_ [GPa]	*ν*	*σ*_y_ [MPa]	*σ*_0.01_ [MPa]	*ε*_y_ [%]	*σ*_B_ [MPa]	*ε*_B_ [%]
0	3.73	0.38	38.43	-	2.65	35.66	4.82
01	2.91	0.352	-	23.66	-	28.14	1.67
0101	2.76	0.402	-	20.2	-	29.13	2.97
0102	2.86	0.396	-	21.69	-	28.87	2.51
0103	2.82	0.397	-	21.03	-	25.85	2.05

## Data Availability

The original contributions presented in the study are included in the article, further inquiries can be directed to the corresponding author.
